# Research biopsies in kidney transplantation: an evaluation of surgical techniques and optimal tissue mass allowing molecular and histological analyses

**DOI:** 10.1186/s12014-024-09508-2

**Published:** 2024-09-14

**Authors:** Sadr ul Shaheed, Hannah McGivern, Marta Oliveira, Corinna Snashall, Chris W. Sutton, Ka Ho Tam, Simon Knight, Syed Hussain Abbas, Jesper Kers, Sarah Cross, Rutger Ploeg, James Hunter

**Affiliations:** 1 Nuffield Department of Surgical Sciences and Biomedical Research Centre, John Radcliffe Hospital, University of Oxford, Headley Way, Headington, Oxford, OX3 9BQ UK; 2 NIHR Oxford Biomedical Research Centre, University of Oxford, Oxford, UK; 3 Bodleian Health Care Libraries, John Radcliffe Hospital, University of Oxford, Oxford, OX3 9DU UK; 4 Institute of Cancer Therapeutics, University of Bradford, Bradford, UK; 5 Institute of Biomedical Engineering, University of Oxford, Oxford, UK; 6 Oxford University Hospital NHS Foundation Trust, Oxford, UK; 7 Department of Pathology, Amsterdam UMC, University of Amsterdam, Amsterdam, Netherlands; 8 Department of Pathology, Leiden Transplant Center, Leiden University Medical Center, Leiden, Netherlands; 9 National QUOD Biobank Consortium with NHS Blood and Transplant, Bradford, UK

**Keywords:** Kidney transplantation, Punch biopsy, Core needle biopsy, Shotgun proteomics

## Abstract

**Background:**

Research biopsies have great potential to advance scientific knowledge by helping to establish predictors of favourable or unfavourable outcomes in kidney transplantation. We evaluated punch and core biopsies of different sizes to determine the optimal size for clinical use.

**Methods:**

A total of 54 punch biopsies and 18 core needle biopsies were retrieved by three transplant surgeons. Each surgeon obtained three separate 2 mm, 3 mm and 4 mm punch biopsy samples and three 23 mm (length) core needle biopsies from two pig kidneys.

**Results:**

4 mm punch biopsies yielded the greatest amount of protein (2.11 ± 0.41 mg) with good reproducibility between surgeons and biopsy types (Coefficient of Variation ∼ 22.13%). All surgeons found 2 mm biopsies technically challenging to obtain and sample processing was difficult due to the sample size. Shotgun proteomics identified 3853 gene products with no significant difference in the quantitative proteome of 2 mm and 3 mm punch biopsies. However, the expression of 158 Kidney enriched genes, was higher in bigger and deeper 4 mm punch and core needle biopsies compared to 2 mm biopsy. Only 80% of 2 mm biopsies demonstrated the presence of glomeruli, whereas glomeruli were present in 100% of all other biopsy sizes.

**Conclusions:**

The 2 mm punch biopsy has been shown to be challenging to use and frequently provides inadequate tissue for histology and proteomics while 3 mm research biopsies were the smallest size that were technically obtainable with adequate tissue for molecular studies.

**Supplementary Information:**

The online version contains supplementary material available at 10.1186/s12014-024-09508-2.

## Introduction

In kidney transplantation research, the quality and quantity of biopsy samples are crucial for conducting accurate molecular and histological analyses. High-quality tissue samples provide a detailed view of the transplanted kidney’s cellular and molecular environment, which is essential for diagnosing rejection, monitoring transplant health, and evaluating therapeutic interventions. Donor kidney biopsies are acquired for both clinical and research purposes and can be obtained using a punch or needle core device. Optimising biopsy techniques is fundamental to advancing our understanding of graft survival and transplant success. The challenge in kidney transplantation research lies in obtaining sufficient tissue for analysis while minimising the risk to the patient. Biopsies must be large enough to yield high-quality samples for multiple technologies, such as genomics, proteomics, and histopathology, yet small enough to reduce complications. Various biopsy techniques, including needle and punch biopsies, have been evaluated to achieve this balance. Needle biopsies, though less invasive and safer, often provide smaller samples that may be inadequate for comprehensive analyses. Punch biopsies can obtain larger samples necessary for in-depth studies but increase the risk of bleeding and other complications [1].

The Quality in Organ Donation (QUOD) biobank was established in 2012 and rolled out in 2014 as a national resource facilitating more in-depth research in organ donation and transplantation in the UK (https://quod.org.uk/). From 2014 to 2018, the QUOD biobank used a core biopsy needle (23 mm throw length) for kidney samples. In 2018, the PITHIA (Pre-Implantation Trial of Histopathology in Renal Allografts) study used a 5 mm punch biopsy to evaluate the role of donor kidney histopathology in organ utilization and clinical decision-making [2]. To align with PITHIA, QUOD also adopted a 5 mm punch biopsy. However, due to bleeding incidents, the biopsy size was reduced to 2 mm in mid-2019, which remains the current protocol. PITHIA also reduced its biopsy size to 4 mm until 2022. Since reducing to a 2 mm punch, retrieval surgeons have reported challenges in obtaining intact biopsies, leading to inadequate samples for molecular analysis in some cases.

Biopsies obtained for research purposes require substantially more tissue than that needed for a diagnostic biopsy, due to the variety of molecular changes [3]. Moreover, decisions regarding optimal care for various disorders rely on an increasing array of molecular tests, including immunohistochemistry, next-generation sequencing, proteomics and metabolomics for which high-quality biological samples are necessary [1, 3–6]. Mass spectrometry (MS)-based proteomics in combination with two dimensional (2D) liquid chromatography (LC) analytical techniques are enabling novel insights into the modulation of particle surfaces by biological environment and subsequent particle-induced cellular responses to endogenous and exogenous indications [7, 8]. Tandem mass tags (TMT) based LC-MS combined with the multidimensional protein identification technology (MudPIT), is a powerful analytical technique for sensitive, multiplexed, and identical peptide/protein quantification in tissue samples with the lowest system error [7, 9]. This study set out to evaluate the donor kidney biopsies obtained by QUOD and investigate the optimal biopsy size required for adequate quality tissue for histological and proteome assessment while upholding patient safety which is of paramount concern.

## Materials and methods

### Sampling and physical characterisation

**Fig. 1:**
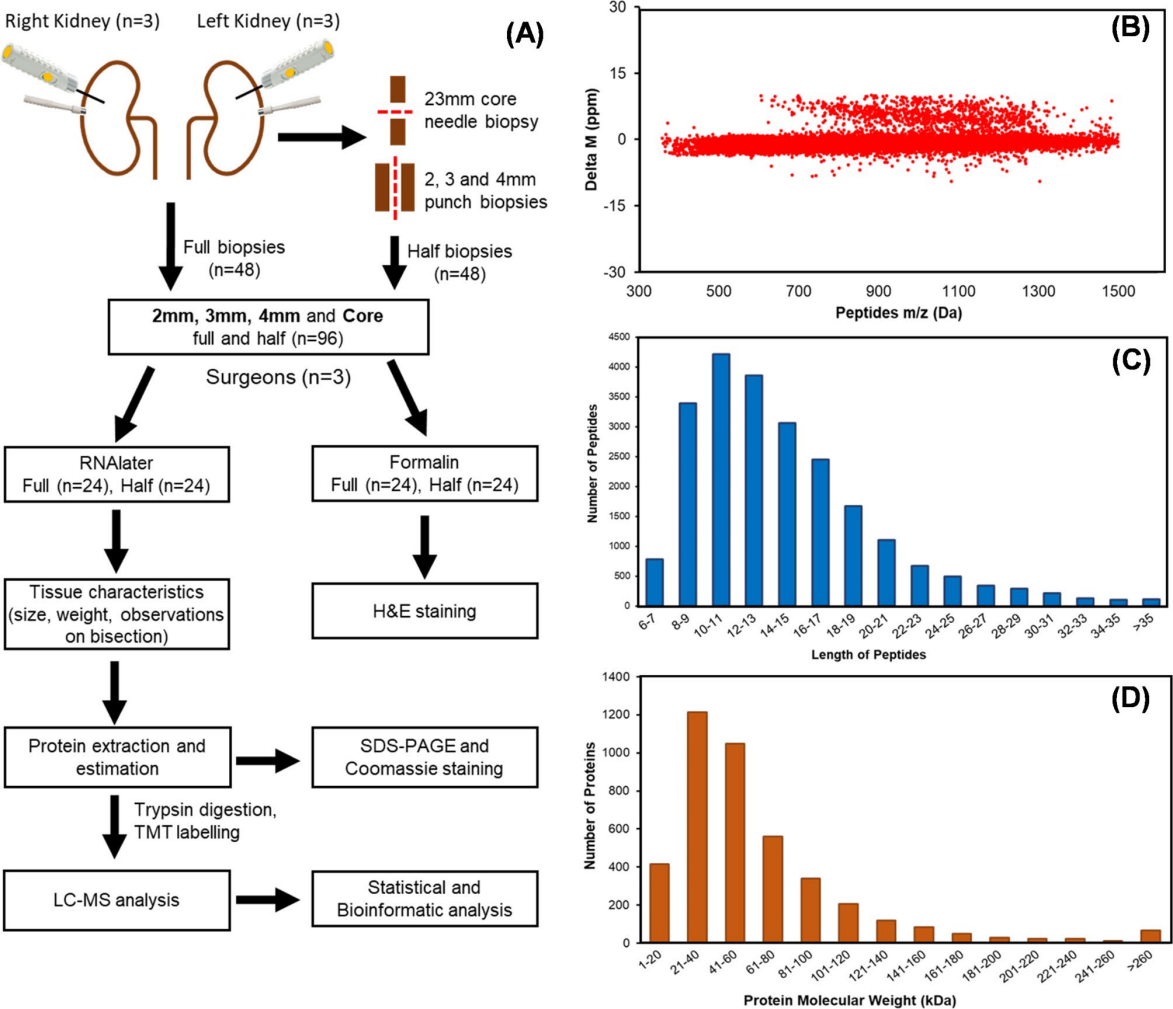
(**A**) Overview of analysis of pig kidney tissues and general workflow for shotgun proteomics workflow coupled with tandem-mass tag (TMT) reagents labelling. One set of punch biopsies (2 mm,3 mm and 4 mm) was bisected longitudinally, while core needle biopsy was split vertically for analysis. (**B**) Mass error distribution of identified peptides. (**C**) Length distribution of peptides. (**D**) Molecular weight distribution of identified proteins

Porcine slaughterhouse kidneys were used for this study, therefore no ethics committee approval was needed. Pigs were slaughtered following Home Office guidance using electrocution and exsanguination via jugular vessels and then they were de-haired in a 60 °C hot wash for 5 min. Kidneys were procured, flushed with Soltran (Baxter, UK) and transferred to the lab using static cold storage. Three transplant surgeons with procurement and QUOD biopsy experience each performed three sets of 2 mm, 3 mm and 4 mm punch biopsies and three 23 mm (throw length) core needle biopsies from two separate pig kidneys. In accordance with standard QUOD practices, one set of kidney biopsies taken by each surgeon was bisected longitudinally, with one half placed in formalin and one half in RNAlater. The other sets of biopsies were left intact and preserved in either formalin or RNAlater. Figure 1A shows a schematic overview of the experimental workflow and examples of the full and half biopsy sizes. A qualitative review of the biopsy technique was recorded by each surgeon following completion of all the biopsies.

### Tissue preparation and histopathological examination

Tissues preserved in 4% formalin were processed into formalin-fixed, paraffin-embedded (FFPE) blocks using standard protocols, as described previously [10]. Tissue sections with a thickness of 4 μm were cut, and subsequently stained using haematoxylin and eosin (H&E). These sections were then scanned using a Glissando Desktop Scanner (Objective Imaging, Kansasville, WI, USA) and analysed by a consultant renal histopathologist, who was blinded to the type of biopsy for each sample to avoid any bias. A score was provided for the following characteristics: biopsy size and quality, number of glomeruli and morphology. Any additional comments from the pathologist concerning tissue quality were also documented.

### Protein assessment and shotgun proteomics

Tissue biopsies preserved in RNAlater and subsequently snap frozen were characterized for size and weight. The protein concentration of each tissue extract was measured using the Pierce™ BCA Protein Assay Kit (Thermo Scientific, Waltham, MA, USA) after extracted by RIPA lysis buffer and each protein extract (∼ 20 μg of protein) was analysed by SDS-PAGE as described previously [7]. The amount of protein recovered from 2 mm biopsies was not enough for shotgun proteomics, so we combined the 2 mm tissue lysates to get ∼ 100ug of material for proteome profiling. A total of 11 samples; 2 mm (*n* = 1, 3 mm (*n* = 4), 4 mm (*n* = 3) and core (*n* = 3), were reduced, alkylated, and digested overnight using Pierce™ Trypsin Protease and labelled with TMT 11-plex reagent according to the manufacturer’s protocol (Thermo Scientific, USA). The TMT-labelled samples were analysed on Dianox ultimate 3000 HPLC system interfaced with a Thermo Orbitrap fusion Mass spectrometer (see Supporting Information Materials and Methods). The MS/MS spectra were processed using Proteome Discover version 2.2, and database searches were carried out against a target and decoy separated Sus scrofa (Pig) database downloaded from Uni-Prot (database version 2021 containing 119,328 protein sequences). Figure 1A Illustrates the standard shotgun proteomics workflow. The mass spectrometry proteomics data have been deposited to the ProteomeXchange Consortium via the PRIDE [11] partner repository with the dataset identifier PXD032354.

### Data analysis and bioinformatics

Proteins identified with one unique peptide and where that unique peptide was identified twice in the analysis, were selected for further analysis. All the statistical analyses were undertaken using Bioconductor Software Package version 3.14, in R Studio version 1.4.1106 (R Studio, Boston, MA) and PRISM version 7.0 software (GraphPad Software, San Diego, CA). Protein abundance was normalised with median approach and converted to log2 for statistical analysis. The over-representation analyses of Gene Ontology (GO) GO terms, including the cellular components, biological process, molecular function, and enriched pathway analysis was performed using FunRich; Functional Enrichment Analysis Tool (www.funrich.org).

## Results

### Qualitative review of biopsy technique and tissue analysis

A total of 54 punch biopsies and 18 core needle biopsies were obtained, with 18 of the punch biopsies and 6 of the core needle biopsies cut in half longitudinally. Therefore, a total of 96 samples were available for analysis (36 full punch biopsies, 36 half punch biopsies, 12 full core biopsies and 12 half core biopsies). All three surgeons reported technical difficulties obtaining 2 mm punch biopsies, including the inability to retrieve the biopsy and damage to the biopsy during procurement. Two biopsies were inadequate and needed to be repeated. In addition, cutting the 2 mm punch biopsy in half longitudinally was frequently not possible due to the small amount of tissue. Preparation for analysis of 2 mm punch biopsies was therefore challenging with occasional damage to tissue and a reduction in usable material.

### Biopsy characteristics and protein assessment

The average biopsy size and total protein recovered from each full-size biopsy are shown in Table 1. The 3 mm and 4 mm punch biopsies had better reproducibility in size across different surgeons compared to the 2 mm punch and core needle biopsies. This is demonstrated by the coefficient of variation (CV) in biopsy size between surgeons. A lower CV indicates more consistent results: the 4 mm punch biopsies had a CV of 22.13%, indicating high reproducibility, while the 3 mm biopsies had a CV of 48.29%. In contrast, the 2 mm punch biopsies had a high CV of 78.64%, reflecting significant variability between surgeons.

**Table 1:** Average biopsy size length x width (mm^2^), coefficient of variation (CV%) between the three surgeons and total protein content (mg) and coefficient of variation (CV%) of protein recovery in the full and half-size 2 mm, 3 mm and 4 mm punch biopsies and the core needle biopsies

Types of biopsies		Size of Biopsy (mm^2^)		Amount of protein (mq)	
		Mean ± SD	CV (%)	Mean ± SD	CV (%)
2mm	Full	4.67 ± 3.67	78.64	0.32 ± 0.13	39.96
	Half	2.50 ± 1.38	55.14	0.19 ± 0.07	39.47
3mm	Full	10.83 ± 5.23	48.29	0.69 ± 0.48	69.09
	Half	6.58 ± 1.62	24.69	0.63 ± 0.29	45.79
4mm	Full	14.50 ± 3.21	22.13	2.11 ± 0.41	19.63
	Half	9.33 ± 2.42	25.95	1.44 ± 0.58	40.73
Core	Full	12.0 ± 9.43	78.57	2.45 ± 0.46	18.96
	Half	6.0 ± 4.0	66.67	0.74 ± 0.25	34.58

**Fig. 2:**
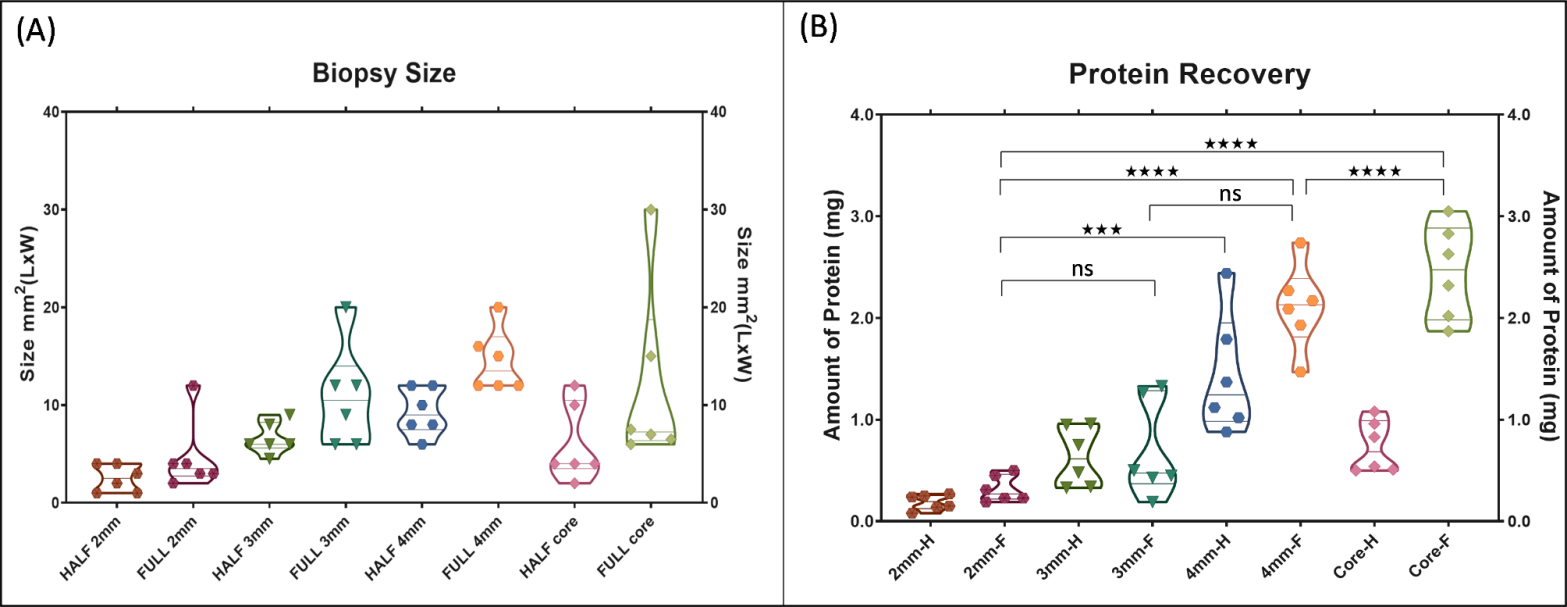
(**A**) Violin plot (truncated) of biopsy size (mm^2^) for the full and half samples of 2 mm, 3 mm and 4 mm punch biopsies and core biopsies. (**B**). Graph showing protein recovery (mg) for the full and half samples of 2 mm, 3 mm and 4 mm punch biopsies and core biopsies. Protein recovery from different tissue biopsies was analysed by Tukey’s multiple comparisons test (ns; not significant, ***; *p* value < 0.0001, ****; *p* value < 0.00001)

Moreover, protein recovery was generally more successful with larger biopsy sizes. The 2 mm punch biopsies often yielded insufficient tissue for effective extraction, while the 3 mm and 4 mm biopsies provided more consistent and adequate tissue, leading to better protein recovery for downstream analysis (Fig. 2B).

### Histology

**Fig. 3:**
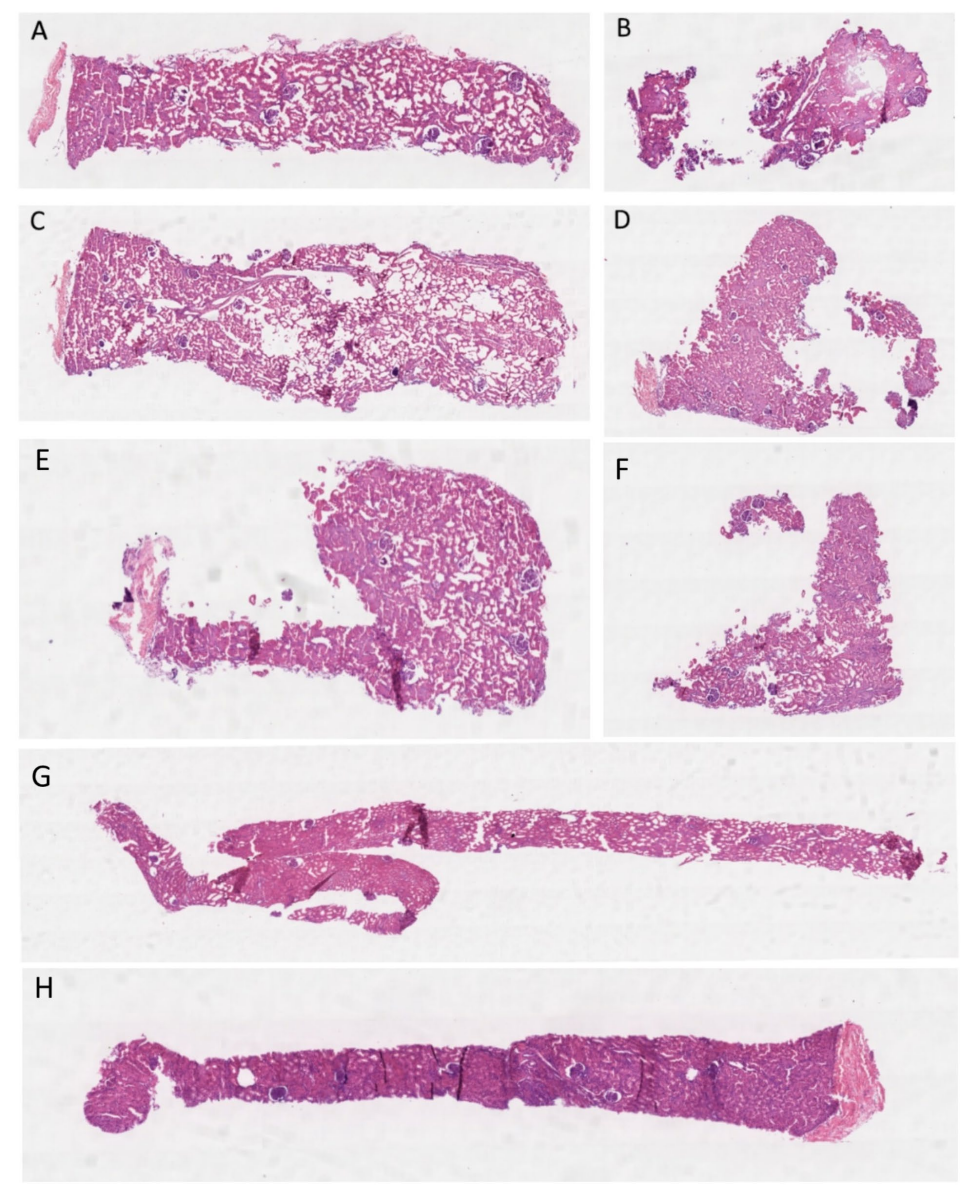
H&E-stained sections of **A**) 2 mm full, **B**) 2 mm half, **C**) 3 mm full, **D**) 3 mm half, **E**) 4 mm full, **F**) 4 mm half, **G**) core full and **H**) core half biopsies. There are more glomeruli present in the 3 mm full (**C**) compared with the 2 mm full (**A**) samples. The 2 mm half biopsy (**B**) is mainly medulla, with no glomeruli present

As the size of the punch biopsy increased, there was a corresponding increase in the number of glomeruli observed, which serves as an indicator of cortical sampling (Fig. 3). The Kruskal-Wallis test revealed no significant difference in the number of glomeruli among the different biopsy groups (*p* = 0.136). Notably, 20% of the full 2 mm biopsies and 25% of the half 2 mm biopsies lacked any glomeruli. In contrast, glomeruli were present in all of the full 3 mm, 4 mm, and core biopsy samples (Table 2). Figure 3 shows the hematoxylin and eosin (H&E) stained sections of the full and half biopsies for the 2 mm, 3 mm, 4 mm, and core samples.

**Table 2:** Total number of glomeruli, the number of glomeruli per biopsy (mean ± SD) and the proportion (%) of biopsy samples with glomeruli for the full and half samples of 2mm, 3mm and 4mm punch and core biopsies

Types of biopsies		Total Glomeruli in all samples	Number of Glomeruli (Mean ± SD)	Tissue sections with Glomeruli (%)
2mm	Full	25	3.12 ± 1.64	80
	Half	21	3.50 ± 1.76	75
3mm	Full	60	5.00 ± 4.02	100
	Half	46	4.60 ± 3.63	85
4mm	Full	45	3.75 ± 1.81	100
	Half	60	5.45 ± 2.84	100
Core	Full	68	6.18 ± 2.89	100
	Half	34	4.25 ± 1.83	100

### Gel electrophoresis

Coomassie Brilliant Blue is commonly used to visualise proteins isolated from complex biological samples, after they have been separated on the basis of size by sodium dodecyl sulphate polyacrylamide gel electrophoresis (SDS-PAGE) [7]. Each tissue lysate (∼ 20 μg of protein) was resolved on SDS-PAGE and stained with Coomassie Brilliant Blue, to check the quality of extracted proteins. There was no qualitative difference in the intensity of Coomassie stained bands, between biopsy size or surgeon, confirming an efficient extraction and purification of proteins from tissues biopsies (see Supporting Information Figure S1).

### Quantitative shotgun proteomics

**Fig. 4:**
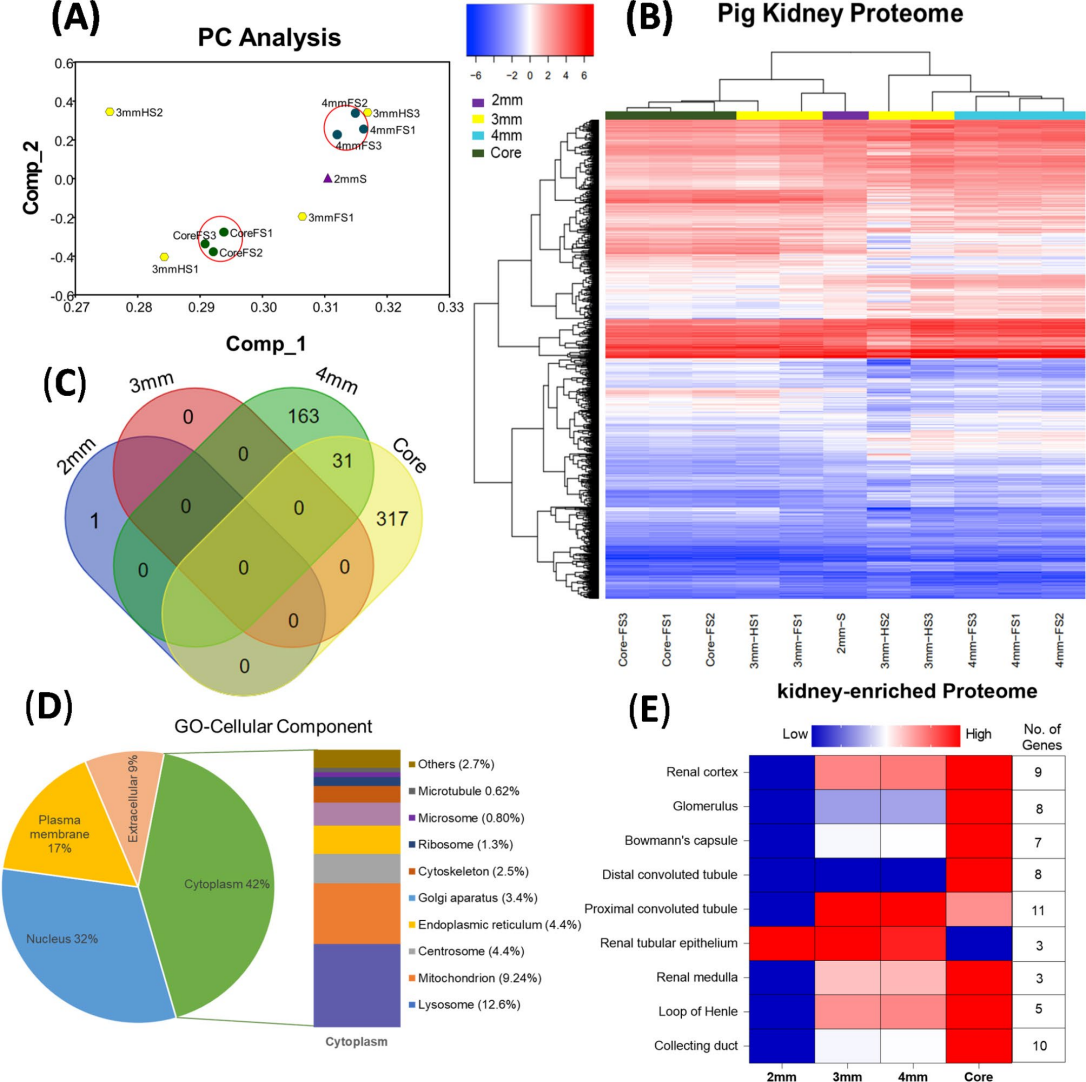
(**A**) The principal component analysis (PCA) was performed on the TMT intensity of 2 mm, 3 mm and 4 mm punch biopsies and core biopsies and samples are represented by individual colours and shapes. (**B**) Hierarchical cluster analysis depicts log2 values of TMT intensity ratios and represented according to the indicated row normalized colour scheme. (**C**) Venn diagram represents significant differentially expressed proteins (3854) in 2 mm, 3 mm, 4 mm and core biopsies groups (Fold change ≥ 1.5; *P* < 0.05). (**D**) Gene-Ontology (GO) cellular components analysis of the Pig Kidney proteome, area of a sector is proportional to the number of genes annotated to the corresponding GO category. (**E**) The expression of proteins specifically enriched in the kidney

To systematically analyse the protein profiles of pig kidneys biopsies, we performed LC-MS analysis (Fig. 1A). Supporting Information Table S1 lists the general quantitative information of the identified proteins. We identified 335,485 spectra in total and obtained the quantitative information of 22,916 peptides and 4193 proteins in tissue biopsies. Among these, 3853 proteins were identified with at least one unique peptide and that unique peptide was identified and quantified in all 11 samples: 2 mm punch (*n* = 1), 3 mm punch (*n* = 4), 4 mm punch (*n* = 3) and core needle (*n* = 3). Among the identified peptides, 86% of peptides had a mass error of ± 2 ppm (part per million), while 16% peptides had mass errors of ± 10ppm (Fig. 1B). The coverage of most proteins ranged from 1 to 50%; the number of proteins with a coverage greater than 10% accounted for 58% of identified proteins (3853). These results showed an acceptable mass accuracy of the MS data. The length of the majority of the peptides ranged from 8 to 21 amino acids (Fig. 1C) which confirmed the complete digestion of the proteins in our sample preparation. Proteins with a molecular weight greater than 100 kDa accounted for 17% (Fig. 1D), providing abundant information for analysis of macromolecular proteins. These results demonstrate the credibility of the proteome profile provided by the TMT-labelled mass spectrometer. Principal components analysis was used as an exploratory technique to investigate clustering of datasets within the high-dimensional space and demonstrated the discrimination between different sample groups; 2 mm, 3 mm, 4 mm and core (Fig. 4A). Full 4 mm and core biopsies were clustered together effectively while 3 mm (full and half) biopsies had poor clustering. Hierarchical cluster analysis of protein expression showed a clearly different expression profile between biopsy types (Fig. 4B). Therefore, the selection of tissue acquisition technique and biopsy size is very important for quantitative comparisons between control and treatment groups in kidney transplantation research. We used an Empirical Bayes method, “LIMMA” for significant differences in expression of identified proteins in each experimental group; 2 mm, 3 mm, 4 mm punch and core needle biopsies. By comparison of 2 mm, 3 mm, 4 mm and core biopsies, there was no significance difference (Fold change ≥ 1.5; *P* < 0.05) in the quantitative proteome of 2 mm and 3 mm punch biopsies, only one protein, PHB domain-containing protein (ERLIN1), was elevated significantly (*P* ∼ 0.01) in 2 mm punch biopsies Fig. 4C, see Supporting Information Table S2). The larger sample obtained from the 4 mm punch had 146 gene products significantly upregulated and 48 downregulated (3.79% and 1.24% of identified 3854 proteins), respectively, and in the core needle biopsy group, 81 proteins (2.1%) were upregulated and 267 (6.9%) were downregulated (Fig. 4C, see Supporting Information Table S2).

### Distribution and functional analysis of cellular components in kidney biopsies

The GO analysis showed that most of the modulated proteins have cytoplasmic origin (42%), followed by nuclear proteins (32%) and then membrane region (17%) (Fig. 4D). Further quantitative analysis of cellular components across different biopsy sizes from kidney tissues indicates that 3 mm and 4 mm punch biopsies generally provide a more representative distribution of cellular components compared to 2 mm biopsies. Specifically, the 2 mm biopsies show disproportionately high percentages in certain areas, such as the cytoplasm, which constitutes 25% of the quantity in 2 mm samples. This percentage increases to 100% in 3 mm samples, 81% in 4 mm samples, and decreases to 58% in core samples. Notably, the 4 mm biopsies exhibit more consistent percentages across different components such as the plasma membrane (100%) and Golgi apparatus (74.21%), suggesting a broader coverage of tissue architecture. In contrast, the 2 mm biopsies often skew towards specific cellular components, making them less reliable for studies requiring a holistic view of cellular morphology.

A functional classification of these proteins revealed that the hydrolase activity, DNA binding, extracellular matrix structural constituent, calcium ion binding and transcription regulator activity was upregulated in 3 mm, 4 mm and core biopsies compared to 2 mm biopsies. Immune response, cell adhesion, cell proliferation, anti-apoptosis and regulation of cell cycle, were the most upregulated biological processes in 4 mm and core groups compared to 2 mm and 3 mm groups. Transcription factors are proteins that regulate the expression of genes by binding to specific DNA sequences [12]. In the pig kidney proteome, seven transcription factors: ZNF238, ZNF513, HNF4A, NFIC, STAT1, CUX1 and Rab-40B RAB40B, were also identified, with elevated expression in 3 mm, 4 mm and core biopsies compared to 2 mm biopsies (see Supporting Information Table S2). We also found 431 proteins with transmembrane domains, with higher expression in 3 mm and 4 mm biopsies compared to 2 mm and core biopsies, indicating wider and deeper biopsies are important for better molecular profiling (see Supporting Information Table S2).

### Identification of kidney-enriched genes and biomarkers for transplantation outcomes

The PIG RNA Atlas database (https://www.rnaatlas.org) contains quantitative annotations of genes expression across the different cell types (e.g. proximal tubule cells, glomerular cells) and we compared our data to identify PIG kidney enriched genes using the FunRich data analysis tool. Total 158 Kidney enriched genes were identified (see Supporting Information Table S3), among those 11 genes were localised to the proximal tubules, 10 genes to collecting ducts and 9 genes to the glomerulus (Fig. 4E). The expression of all these kidney enriched genes was higher in 4 mm and core groups compared to the 2 mm group (Fig. 4E). We also identified 22 genes products from the major facilitator (TC 2.A.1) superfamily in the kidney, among those 10 genes are ether specific or highly enriched in kidney tissues such as SLC5A2, SLC5A12, SLC6A18, SLC6A19, SLC17A1, SLC22A1, SLC22A2, SLC25A15, SLC27A2 and SLC34A1. All these genes had higher expression in 3 mm and 4 mm biopsies compared to the 2 mm biopsy (see Supporting Information Table S2). The highly enriched genes SERHL2 and HSD11B2, in renal cortex were significantly upregulated (> 1.5-fold, p. value < 0.05) in 4 mm punch biopsies compared to core needle biopsies. The expression of renal medulla enriched genes, AQP2 and FXYD2, was significantly higher (> 1.5-fold, p. value < 0.05) in core need compared to punch biopsies. So, 4 mm punch biopsy are better representation of renal cortex, while core needle biopsy can be for renal medulla.

Potential biomarkers in the context of ischemia reperfusion injury and delayed graft function were also identified in pig kidney proteome, such as Galectin-3, Annexin A2, Endoglin and BH3 interacting domain death agonist [13]. We also identified a number of proteins being investigated as post-transplant indicators for acute rejection: Peptidyl-prolyl cis-trans isomerase FKBP5, complement factor D (CFD), Pigment epithelium-derived factor (SERPINF1), Nicotinamide phosphor-ribosyl transferase, Mitogen-activated protein kinase 9, GTP-binding protein Rheb, Proteasome subunit beta 9 (PSMB9) and Brain acid soluble protein 1 (BASP1) [13].

## Discussion

This study focused on an evaluation and optimisation of both surgical technique and best size for quality of kidney biopsies obtained for research from donor kidneys prior to transplantation whilst keeping the risk of complications minimal. All three surgeons reported that obtaining the 2 mm biopsies was challenging and it was frequently not possible to cut longitudinally due to the small fragile amount of tissue obtained. These findings were consistent with previous reports to QUOD from procurement surgeons who had difficulty retrieving 2 mm biopsies. There have also been reports from researchers who have received 2 mm punch biopsy samples from the QUOD biobank, that the tissue obtained was too small for analysis, which is consistent with the results from this study.

Overall, 4 mm punch biopsies yielded the largest amount of tissue, the least variability in size and weight of biopsy and the most optimal sample for histological analysis. However, the 4 mm punch resulted in the largest defect in the kidney. 3 mm punch biopsies yielded adequate tissue for proteomic analysis and 100% of full biopsy samples demonstrated a presence of glomeruli on histological assessment. This was in contrast to 2 mm biopsies which had glomeruli present in 80% of samples. Interestingly, the needle core biopsy always contained sufficient glomeruli and was therefore equivalent to the 4 mm punch in quality for histological assessment. However, the variation of tissue size and weight from the needle core was poor, at 78.57%, which was similar to that of the 2 mm punch with a coefficient of variation of 78.64%. Despite the poor correlation between surgeons, the tissue mass obtained by the needle core was excellent at 2.45 mg compared with 0.32 mg for the full 2 mm punch biopsies.

Shotgun proteomics, utilising multidimensional protein identification technology, can enable identification of the specific proteomes of different functional units, such as glomeruli and tubules [14–18]. Normally, 3–20 mm size biopsies are required at starting point to isolate these different components of kidney for multi-omics profiling [15–17]. For post translation modifications of peptides and proteins, such as phospho-proteomic, 0.45-1000 mg of tissue lysate is required to get satisfactory research data [19, 20]. The amount of protein recovered from 2 mm biopsy size was enough for individual assays such as SDS-PAGE, immunoblotting or discovery proteomics, but not discovery and validation proteome assessment. 3 mm, 4 mm and core biopsies gave enough material for discovery proteomics and also for clinical validation of discovery targets (Table 1). The expression of kidney specific proteome was higher in 3 mm, 4 mm punch and core needle biopsies, indicating larger biopsies are required to enable an in-depth characterisation of the kidney tissue.

The main limitation of this study is that it was experimental and used pig rather than human tissue. However, pig kidney is readily available, anatomically and structurally similar to the human kidney and is considered an appropriate alternative [21]. We also did not assess the ease of suturing the different sized defects and none of the kidneys were transplanted to test the difference in haemostasis following biopsy repair. However, the rates of complications following biopsy are well documented and NHSBT data have shown that despite changing from 2 mm to 3 mm the serious adverse event rate of less than 0.09% has remained unchanged. Interestingly, during the PITHIA trial there were no reported serious adverse events from the 4–5 mm punch biopsy [2].

The core needle biopsy was included as part of the audit as it was the previous gold standard and is still widely used. Limitations of the core needle technique include poor reproducibility of tissue biopsy and complicated molecular profiling. Tissue samples are inconsistent and often contain a larger proportion of medulla compared to cortex and this is particularly problematic when the biopsy is halved, as is standard QUOD practice. Complications such as arteriovenous fistula and injury to the collecting system are well documented in the literature and were previously reported to QUOD [22, 23]. This resulted in the switch from needle core to punch biopsy and is why it remains the more favourable of the two options. The 2 mm punch biopsy has been shown to be challenging to use and frequently provided inadequate tissue for histology and proteomics. Given the QUOD biopsy is obtained purely for the purposes of research, the 2 mm has been shown to be insufficient. Increasing the punch biopsy size from 2 mm to 3 mm will minimise the challenges faced by retrieval surgeons in obtaining the biopsy and provide adequate tissue for research without increasing the risk to the recipient.

## Electronic supplementary material

Below is the link to the electronic supplementary material.

Supplementary Material 1

Supplementary Material 2

Supplementary Material 3

Supplementary Material 4

## Data Availability

The mass spectrometry proteomics data have been deposited to the ProteomeXchange Consortium via the PRIDE partner repository with the dataset identifier PXD032354.
