# Supplementary material for: Research biopsies in kidney transplantation: an evaluation of surgical techniques and optimal tissue mass allowing molecular and histological analyses
Source: Clin Proteomics. 2024 Sep 14;21:55. doi: 10.1186/s12014-024-09508-2 (PMC11401365; doi:10.1186/s12014-024-09508-2)
Supplement: Supplementary file 4 — Supplementary Material 4 [file 12014_2024_9508_MOESM4_ESM.docx]

**Supplementary Data**

**RESEARCH BIOPSIES IN KIDNEY TRANSPLANTATION: AN EVALUATION OF SURGICAL TECHNIQUES AND OPTIMAL TISSUE MASS ALLOWING MOLECULAR AND HISTOLOGICAL ANALYSES**

# Material and Methods

## Protein Assessment and Digestion

Tissue biopsies preserved in RNAlater and subsequently snap frozen were characterized for size and weight. Protein was extracted from tissue biopsies using RIPA lysis buffer (Merck & Co, Kenilworth, NJ, USA) containing phosphate inhibitor and protease inhibitor cocktail. 3x volume of RIPA lysis buffer was added to tissue biopsies, which were vortexed for 2 minutes at room temperature and then sonicated for 20 seconds on ice using a Status US70 sonicating probe (Philips Harris Scientific, UK). The samples were centrifuged at 13,400 rpm for 25 minutes at 4°C and the liquid phase extracted to new tubes. The protein concentration of each biopsy extract was measured using the Pierce™ BCA Protein Assay Kit (Thermo Fisher Scientific, Waltham, MA, USA) according to manufacturer guidelines, and remaining extracts were stored at -20^o^C. ~20ug of each protein was analysed by Sodium dodecyl sulphate-polyacrylamide gel electrophoresis (SDS-PAGE) followed by Coomassie blue staining.

Protein enrichment was performed overnight using 8 volumes of 100% acetone at -20^o^C. Samples were centrifuged for 20 minutes at 13,400 rpm at 4^o^C and the pellet (~100 μg of protein) resuspended in 0.1M Triethylammonium bicarbonate (TEAB) (Merck & Co, Kenilworth, NJ, USA) with 1% w/v SDS. Each protein sample was reduced with 0.5M TCEP at 37 °C for at least 1 h followed by alkylation with 40 mM iodoacetamide (IAA) for 30 minutes in the dark. The protein solution was diluted 1:5 with 0.1M TEAB and digested with 0.5 μg/μL Pierce™ Trypsin Protease, MS Grade (Thermo Fisher Scientific, Waltham, MA, USA) at 37°C overnight, followed by stopping the reaction with formic acid. Digests were desalted on SPE Cartridge Bond Elut C18 columns (Agilent Technologies, Santa Clara, CA, USA), and peptides were eluted with 60% acetonitrile, then lyophilized via vacuum centrifugation.

## Tandem Mass Tags Labelling and High-Performance Liquid Chromatography Fractionation

Equal amounts of desalted peptides (100μg) from each sample were reconstituted in 100 mM TEAB (Merck & Co, Kenilworth, NJ, USA) and labelled with TMT 11-plex reagent according to the manufacturer’s protocol for the TMT Kit (Thermo Fisher Scientific, Waltham, MA, USA). TMT-labelled peptides were combined and lyophilized by vacuum centrifugation.

Before LC-MS/MS analysis, TMT-labelled samples were pre-fractionated on Agilent 1260 Infinity II high performance liquid chromatography instrument (Agilent Technologies, Santa Clara, CA, USA) system, to reduce their complexity. Total of 24 fractions were collected after loading 100ul of sample on a XBridge BEH130 C18 column (250mm × 4.6mm; particle size: 5μm). The mobile phases were as follows: Solvent A consisted of 15 mM ammonium hydroxide in 2% acetonitrile and solvent B consisted of 15 mM ammonium hydroxide in 90% acetonitrile. After equilibration, a linear gradient was started as follows: 0–35% solvent B for 35 minutes; 5 minutes; 50–90% solvent B, 4 minutes; column wash with 90% solvent B, followed by 8 minutes column equilibration at flow rate of 0.2 mL/minute. A total of 24 fractions were collected and combined into 16 fractions by keeping fractions 1-12 and combining the fractions 13,14,15 and 16, 17, 18 and, 19, 20, 21 and 22, 23, 24. The combined fractions were dried, lyophilized, and stored at −80°C until LC-MS/MS analysis.

## Reversed-Phase Liquid Chromatography–Tandem Mass Spectrometry

The LC-MS/MS analysis was performed using an Dianox ultimate 3000 HPLC system (Thermo Fisher Scientific, Waltham, MA, USA Thermo Fisher) interfaced with a Orbitrap fusion Mass spectrometer (Thermo Fisher Scientific, Waltham, MA, USA Thermo Fisher). The labelled peptides were resuspended in 20μL solvent A (2% ACN, 0.1% FA), and loaded onto a trap column with 5μm silica particles, 100 Å pore size. After 4 minutes, samples were loaded onto a 25cm analytical column (Thermo Fisher Scientific, Waltham, MA, USA). Peptides were eluted with a 65-minute linear gradient: 2–25% solvent B (90% ACN, 0.1% FA), 10 minutes; 25–50% solvent B, 5 minutes; 50–90% solvent B, 8 minutes; column wash with 90% solvent B, followed by 16 minutes column equilibration with solvent A at a flow rate of 300 nL/min.

The Orbitrap Fusion mass spectrometer was operated in data-dependent mode (DDA). The MS1 survey scan was from 350–1500 m/z, and data was acquired at a high resolution of 120,000 (m/z 200). The target value was 3 × 105 with a maximum ion injection time of 100ms. As for the second stage of mass spectrometry (MS2) scans, charge state; 2-7, Dynamic exclusion; 50 seconds, Cycle time; 3 seconds, were selected from the first stage of mass spectrometry (MS1) full scan with an isolation width of 1.6 m/z from fragmentation in collision induced dissociation~ 35%. Automated Synchronous Precursor Selection (SPS) for MS3 setting was used for quantification of TMT-11PLEX, a special feature of Orbitrap fusion which improves quantitative accuracy when using isobaric mass tags. All MS3 was performed on Orbitrap at 30000 resolutions, scan range 100-500 m/z, maximum injection time 105ms and with 65% high-energy collisional dissociation (HCD). For the setting of the nano electrospray ion source, the spray voltage was 2.2kV; no sheath gas flow; and the heated capillary temperature was 275°C.

## Data Processing and Database Searching

MS/MS spectra were processed using Proteome Discover version 2.2 (Thermo Fisher), and database searches were carried out against a target and decoy separated Sus scrofa (Pig) database downloaded from Uni-Prot (database version 2021 containing 119,328 protein sequences). Trypsin was chosen as the enzyme, allowing up to two missed cleavage sites. The MS1 mass tolerance was set to 10ppm while fragment mass tolerance for MS/MS spectra was set to 0.6 Da. Carbamidomethylation of Cystine (C) and TMT 6-plex (lysine and N-terminus of peptides) were chosen as a static modification. Oxidation of Methionine was specified as dynamic modification. Only rank 1 peptides and Mascot score ≥21 (p. value< 0.05) was accepted and included in further analysis.

# Supplementary Figure: 1

Figure S1. SDS-PAGE analysis of biopsy proteins. 20µg of extracted proteins from each sample was separated by molecular weight using Sodium dodecyl sulphate-polyacrylamide gel electrophoresis (SDS-PAGE) and stained with Coomassie blue dye.
